# Spatio-Temporally Restricted Expression of Cell Adhesion Molecules during Chicken Embryonic Development

**DOI:** 10.1371/journal.pone.0096837

**Published:** 2014-05-07

**Authors:** Priti Roy, Amitabha Bandyopadhyay

**Affiliations:** 1 Department of Biological Sciences and Bioengineering, Indian Institute of Technology Kanpur, Kanpur, U.P., India; Texas Tech University, United States of America

## Abstract

Differential cell adhesive properties are known to regulate important developmental events like cell sorting and cell migration. Cadherins and protocadherins are known to mediate these cellular properties. Though a large number of such molecules have been predicted, their characterization in terms of interactive properties and cellular roles is far from being comprehensive. To narrow down the tissue context and collect correlative evidence for tissue specific roles of these molecules, we have carried out whole-mount *in situ* hybridization based RNA expression study for seven cadherins and four protocadherins. In developing chicken embryos (HH stages 18, 22, 26 and 28) cadherins and protocadherins are expressed in tissue restricted manner. This expression study elucidates precise expression domains of cell adhesion molecules in the context of developing embryos. These expression domains provide spatio-temporal context in which the function of these genes can be further explored.

## Introduction

Regulated cell-cell adhesions control a diverse range of morphogenetic events including migration of cells, separation or fusion of tissues, formation of tissue boundaries, epithelial to mesenchymal transitions or mesenchymal to epithelial transition and establishment of synapses between neurons. Thus it is not surprising that the process of cell adhesion is tightly regulated. A family of cell adhesion molecules, called the cadherins, is particularly important regulator of controlled cell adhesion [1]. The first few members of cadherin family were originally identified as cell surface glycoproteins mediating Ca^++^-dependent homophilic interaction between cells [2]. A number of members (>100) have so far been identified in the cadherin family based on sequence homology and genomic analysis. These members differ in the protein structure. However the characteristic extracellular cadherin repeat is conserved in all of them. Six broad categories of cadherins, including classical cadherins and protocadherins, have been defined based on phylogenetic studies [3].

### 1.1 Cadherins

The classical cadherins are single pass trans-membrane proteins having divergent extracellular domains with five cadherin-type repeats and a conserved cytoplasmic domain [4]. The extracellular regions mediate specific cell-cell interactions. Cadherins expressed on the surface of a cell interact with similar molecules expressed on other cells. These homophilic interactions mediate cell sorting, create cellular sheets like germ layers, epithelia etc., as well as maintain their integrity. Mutations in certain cadherins, E-cadherin and C-cadherin, have been shown to interrupt gastrulation in frog [5] and fish [6] respectively.

The intracellular regions of cadherins interact with a number of cytoplasmic proteins, the best characterized of which are catenins like β-catenin, plakoglobin and p120 [7]. Through interaction with cytosolic proteins and cytoskeletal elements cadherins co-ordinate a large range of cellular functions including cytoskeletal reorganization and signal transduction. Cadherins have been reported to modulate a number of signaling cascades [8] including the MAPK signalling [9], Fgf signalling [10], [11] and VEGF signalling [12], [13].

### 1.2 Protocadherins

Protocadherins are a subfamily of cadherins. Sano *et*. *al*., reported them for the first time [14]. Like classical cadherins, protocadherins also have extracellular domains containing cadherin-type repeats; however, the number of repeats is more than five. Further, the cytoplasmic domains bear no significant homology to classical cadherins. Later on it was found that protocadherins have variable cytoplasmic domains which do not interact with the catenins instead interact with a range of yet to be fully characterized set of proteins including the Fyn-kinases [15], adaptor protein Disabled 1 [16] and protein phosphatase 1 [17]. Few protocadherins have been functionally characterized in the context of Xenopus and Zebrafish embryogenesis. The Xenopus NF-protocadherin (NFPC) was seen to be expressed in the deep and sensorial layers of the ectoderm and epidermis [18]. Expression of a mutant form, where the extracellular domain was deleted, resulted in ectodermal and epidermal lesions thus implicating it in ectodermal differentiation and/or maintenance. Kuroda *et al* isolated the axial protocadherin (AXPC) from Xenopus [19] and through gain of function and loss of function studies demonstrated its role in prenotochordal cell sorting. Similarly, Kim *et al,* isolated the Xenopus paraxial protocadherin (PAPC) from the presomitic mesoderm [20]. Later on the same group proved that during Xenopus somitogenesis, the segmental boundaries are established by PAPC [21]. Recently, protocadherins have been intricately linked to an important cellular behavior exhibited by neuronal processes or dendrites called self-avoidance [22]. It refers to the property of axonal or dendritic projections to spread and cover large spatial territories without crossing its own branches. This is speculated to avoid redundancy and provide better coverage. Lefevbre *et al* reported the role of protocadherins in dendritic self-avoidance [23]. They observed disruption of dendritic self-avoidance in retinal starburst amacrine cells and purkinjee cells upon deleting the mouse *Pcdh* γ-subcluster comprising of 22 *Pcdh* genes. Though studied mostly in the context of nervous system, protocadherins are reported to be expressed in other cell types as well.

It is important to note that homophilic interactions seem to dominate the interactions between cadherins. However they are also reported to participate in heterophilic interactions. As reported by Cepek *et*. *al*., E-cadherins on epithelial cells are involved in heterophilic interactions with α^E^β_7_ integrin expressed by T-lymphocytes [24]. Similarly, Biswas *et*. *al*., [25] and Emond *et*. *al*., [26] have shown interaction between protocadherin-19 and N-cadherin as a novel mechanism of cell adhesion regulating cell movements during anterior neurulation. Further, the same cell type expresses many different cadherins and thus different types of interactions are likely to mediate different aspects of cadherin functions. A detailed study of expression patterns of different members of cadherin family would thus help in appreciating the range of interactions these molecules are engaged in.

Although cadherins were originally named after the tissue in which they were found most prominently, it is now well established that most cadherins are expressed in many more spatio-temporal domains than originally appreciated. During embryogenesis, different tissues are sculpted through series of morphogenetic events. The tissue architectures thus defined are largely maintained throughout life. Thus it is important to study the tissue specific dynamics of cadherin expression during its formation. A comprehensive study reporting the expression patterns of cadherins or protocadherins during the embryonic development of any organism is awaited. Such data can provide important correlative evidence for the roles of cadherins in mediating specific morphogenetic events. For this purpose, we carried out whole mount RNA *in situ* hybridization (WM-ISH)-based screen at four distinct stages of early chick embryonic development (*Hamburger–Hamilton* stages 18, 22, 26 and 28 or HH18, HH22, HH26 and HH28) [27] encompassing most of the early morphogenetic events. With emerging novel cellular roles and interactive properties of cadherins, WM-ISH-based expression data would provide the precise spatial context to speculate and explore the same. A random set of cadherins, including two classical cadherins, five unconventional cadherins and four protocadherins was used for the screen.

## Materials and Methods

### 2.1 Tissue Harvesting and Processing

Fertilized white leghorn chicken (*Gallus gallus*) eggs were procured from Government Poultry Farm, Chak Gazaria, U.P., Lucknow, CSA University of Agriculture & Technology, Kanpur and Santosh’ Poultry Farm, Nankari village, Kanpur. The eggs were incubated in a humidified chamber at 38°C for different durations to get desired stage of development (69 Hrs. for HH18, 3.5 days for HH22, 5 days for HH26 and 6 days for HH28). Embryos were harvested and fixed overnight in 4% paraformaldehyde (Sigma). For WM-ISH, the tissues were dehydrated through methanol (Merck) gradient and stored in 100% methanol at −20°C till further use. For probe synthesis we acquired following chick expressed sequence tags (ChESTs) available with MRC Gene Service (now known as Source Bioscience): ChEST 624i13 for *Cdh1*, ChEST 374k4 for *Cdh2*, ChEST 699f3 for *Cdh4*, ChEST 724e13 for *Cdh5*, ChEST 712b21 for *Cdh11*, ChEST 677d12 for *Cdh13*, ChEST 597c19 for *Cdh23*, ChEST 605d23 for *Pcdh1*, ChEST 653n9 for *Pcdh8*, ChEST 665n5 for *Pcdh12*and ChEST 647e21 for *Pcdh18*.

### 2.2 *In situ* Hybridizations

Whole-mount *in situ* hybridizations (WM-ISH) were carried out as previously described [28] with minor modifications. All WM-ISH were carried out in 12-well plates (CLS3512, Sigma-Aldrich) containing Net-well inserts (CLS3477, Sigma-Aldrich) and holders (CLS320, Sigma-Aldrich). For each gene, two embryos each of HH18, HH22, HH26 and HH28 were used. An expression pattern was recorded if the signal was identical in both the embryos of the same stage. DIG-labeled probes (single detection) were detected with NBT and BCIP (Roche). Templates for anti-sense RNA probe synthesis were generated by polymerase chain reaction with T3 and T7 primers on ChEST clones. All probes were synthesized with T3 RNA polymerase (Promega) and digoxigenin labeled nucleotides (Roche). Stained whole embryos were viewed with Leica DMS6D and images were captured with Leica DFC290.

## Results

### 3.1 *Cadherin 1*

**Figure 1:**
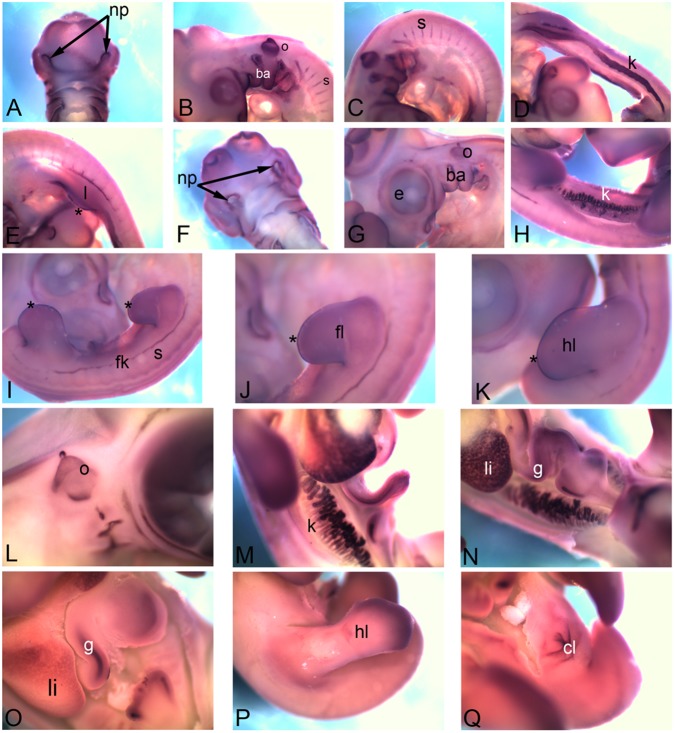
mRNA expression profile of *cadherin 1*. Expression of *Cdh1* was detected at HH18 in nasal primordia (A), branchial arches, otic vesicle (B), somites (C), kidney (D), in and around limb (E), at HH22 in nasal primordia (F), in and around eye, branchial arches, otic vesicle (G), kidney (H), flank (I), forelimb (J), hindlimb (K), at HH26 in otic vesicle (L), kidney (M), liver and gut tube (N), at HH28 in gut tube (O), hindlimb (P) and cloaca (Q). np, nasal primordia; ba, branchial arch; o, otic vesicle; s, somite; k, kidney; l, limb; fk, flank; fl, forelimb; hl, hindlimb; li, liver; g, gut; cl, cloaca; asterisk, apical ectodermal ridge (AER).

*Cadherin1*(*Cdh1*), also known as epithelial cadherin is expressed in the nasal pit (np in Fig. 1A), otic vesicle (o), branchial arches (ba) (Fig. 1B), intersomitic boundary (s in Fig. 1C) intermediate mesoderm (k in Fig. 1D) and in the apical ectodermal ridge (asterisk) within forelimb (l in Fig. 1E) at HH18. At HH22 the nasal pit (np in Fig. 1F) and branchial arch (ba) expression gets more restricted (Fig. 1G). At HH22 nephric duct of the kidney (k), as well as the kidney tubule epithelia are marked by *Cdh1* expression (Fig. 1H). At this stage though the expression in the somites (s) is reduced, another thin band of expression comes up in the flank (fk in Fig. 1I). Expression is distinctly detected in the apical ectodermal ridge (asterisk) of the limb bud (Fig. 1I–1K). At HH26, the otic vesicle (o) expression (Fig. 1L) as well as the kidney (k) tubule expression (Fig. 1M) pattern becomes distinct. Expression is also detectable in the liver (li) and gut (g) (Fig. 1N). At HH28, in most of these locations the abundance of *Cdh1* mRNA seems to go down significantly. It can still be detected in the liver (li) and the gut (g) (Fig. 1O), in the distal periphery of the hindlimb (hl in Fig. 1P) and in cloaca (cl in Fig. 1Q) at very low levels.

### 3.2 *Cadherin 2*

**Figure 2:**
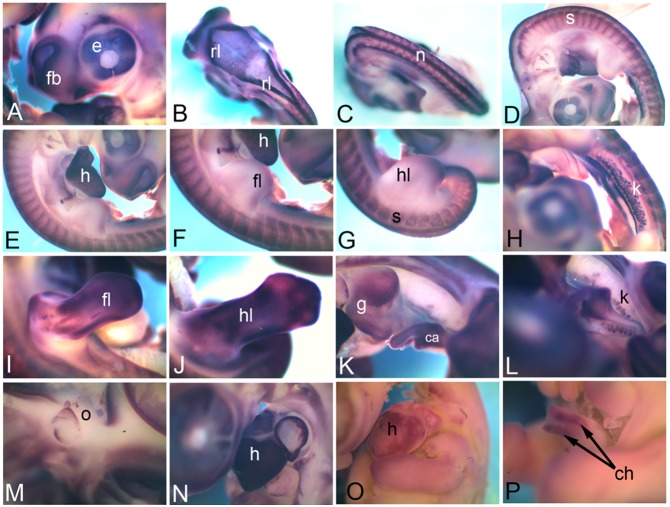
mRNA expression profile of *cadherin 2.* Expression of *Cdh2* was detected at HH22 in forebrain, in and around eye (A), in rhombic lip (B), in neural tube (C), somites (D), Heart (E), forelimb (F), hindlimb (G), in kidney (H), at HH26 in forelimb (I), hindlimb (J), gut tube (K), in kidney (L), otic vesicle (M), heart (N), at HH28 in heart (O) and caecum (P). fb, forebrain; e, eye; rl, rhombic lip; n, neural tube; s, somite; h, heart; hl, hindlimb; k, kidney; fl, forelimb; hl, hindlimb; g, gut; o, otic vesicle; ca, caecum; ch, caecal horns.

No expression of *Cadherin 2* (*Cdh2*) or neural cadherin mRNA could be detected at HH18. Our data suggests that HH22 onwards *Cdh2* is expressed at a low level in a broad range of tissues with certain structures expressing high level of this mRNA. At HH22, expression was detected in the forebrain (fb), rhombic lip (rl), neural tube (n), in and around the eyes (e), heart (h), somites (s), limb buds (fl, forelimb and hl, hindlimb) and kidney (k) (Fig. 2A–H). By HH26, expression domain in the limb buds (fl, forelimb and hl, hindlimb) became broad (Fig. 2I,J); it is also seen in the superficial layers of the gut tube (g) extending till the caecum (ca in Fig. 2K), and in the renal glomeruli within kidneys (k) (Fig. 2L). The expression in the renal glomeruli is more appreciable towards the caudal end (Fig. 2L). At HH26 the otic vesicle (o) expression gets restricted and becomes very distinct (Fig. 2M). It is also detected in the heart (h in Fig. 2N). At HH28 it can be detected in the heart (h), and in the caecal horns (ch) within the caecum (Fig. 2O,P).

### 3.3 *Cadherin 4*

**Figure 3:**
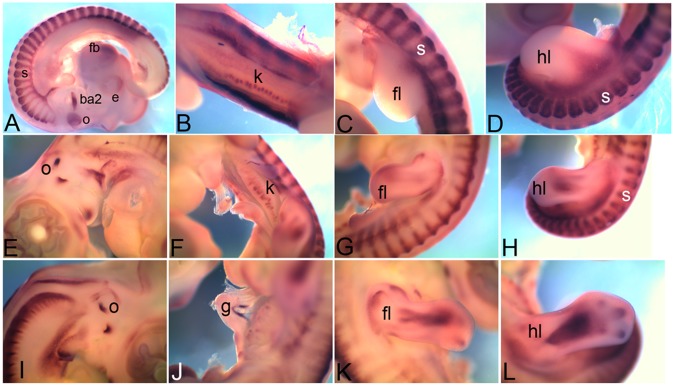
mRNA expression profile of *cadherin 4.* Expression of *Cdh4* detected at HH18 in second branchial arch, otic vesicle, eye, forebrain, somites (A), at HH22 in kidney (B), somite, forelimb (C), hindlimb (D), at HH26 in first otic vesicle (E), kidney (F), in forelimb (G), hindlimb (H), at HH28 in otic vesicle (I), gut (J), forelimb (K), hindlimb (L). ba2, second branchial arch; e, eye; o, otic vesicle; s, somite; fb, forebrain; k, kidney; fl, forelimb; hl, hindlimb; g, gut.

*Cadherin 4* (*Cdh4*) also known as retinal cadherin is expressed in very restricted domains at all stages. At HH18 it is expressed in otic vesicle (o) and in the second branchial arch (ba2) while low level of expression can also be detected in the retina within eye (e) and forebrain (fb) (Fig. 3A). By HH22, expression is also detected in the renal glomeruli within kidney (k) (Fig. 3B), in the forelimb (fl) and hindlimb (hl) and somites (s) (Fig. 3C,D). At this stage *Cdh4* expression within somites (s) is detectable in both myotome and dorsal root ganglia (Fig. 3C,D). By HH26 the expression can also be detected within otic vesicle (o) in endolymphatic duct (Fig. 3E). The expression within kidney gets less distinct (k) while limb bud expression (fl, forelimb and hl, hindlimb) becomes more distinct (Fig. 3F–H). By HH28, the otic vesicle (o) expression gets restricted to segments of endolymphatic duct (Fig. 3I). In gut (g) *Cdh4* expression is detected in a band of tissue on the dorsal side of midgut (Fig. 3J). The expression domain within limb (fl, forelimb and hl, hindlimb) also becomes very distinct (Fig. 3K,L) at this stage.

### 3.4 *Cadherin 5*

**Figure 4:**
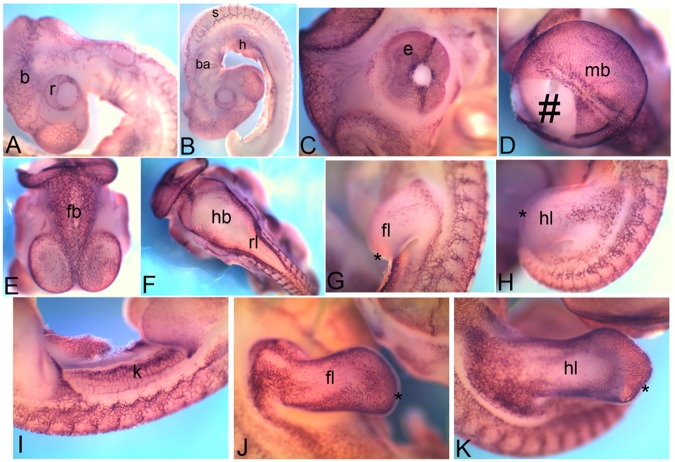
mRNA expression profile of *cadherin 5*. Expression of *Cdh5* at HH18 in brain, retina (A), somites, heart, branchial arch (B), at HH22 in eye (C), midbrain (D), forebrain (E), hindbrain and rhombic lip (F), forelimb (G), hindlimb (H), kidney (I), at HH26 in forelimb (J) and hindlimb (K). b, brain; r, retina; ba, branchial arch; h, heart; s, somite; e, eye; mb, midbrain; fb, forebrain; hb, hindbrain;rl, rhombic lip; fl, forelimb; hl, hindlimb; k, kidney; hash, tissue lost during embryo processing, aterisk, apical ectodermal ridge (AER).

*Cadherin 5*(*Cdh5*) is also known as vascular endothelial cadherin. The overall expression appears like a mesh (Fig. 4). At HH18 *Cdh5* mRNA is detected in the brain (b), retina (r) (Fig. 4A), somites (s), branchial arches (ba) and heart (h) (Fig. 4B). At HH22 it is detected in and around the eyes (e) (Fig. 4C), in midbrain (mb in Fig. 4D), forebrain (fb in Fig. 4E), hindbrain (hb) and rhombic lip (rl) (Fig. 4F) and can also be detected in the limb bud (fl, forelimb and hl, hindlimb in Fig. 4G,H)) and kidney (k in Fig. 4I). At HH26 it continues to be detected in the limbs (fl, forelimb and hl, hindlimb in Fig. 4J,K). It should be noted that in the limb bud, the ectoderm never expresses *Cdh5* (asterisk in Fig. 4G,H,J, K).

### 3.5 *Cadherin 11*

**Figure 5:**
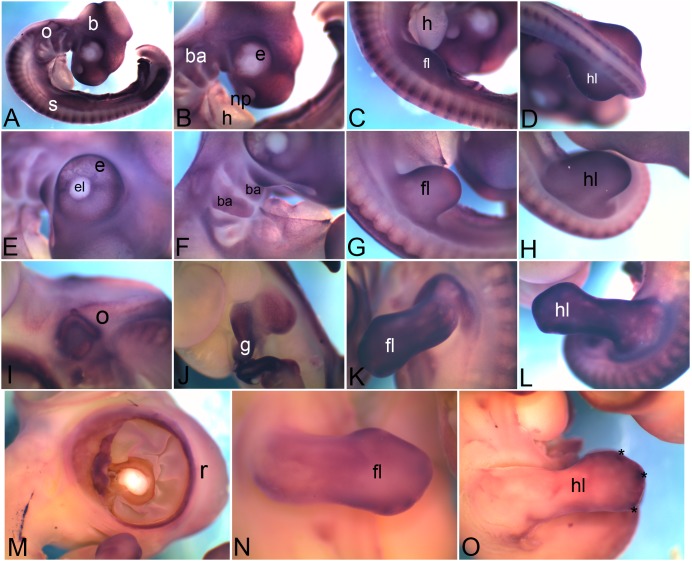
mRNA expression profile of *cadherin 11*. Expression of *Cdh11* at HH18 in brain, somite, otic vesicle (A), branchial arch, eye, nasal primordia, heart (B), forelimb (C), hindlimb (D), at HH22 in and around eye excluding lens (E), branchial arches (F), forelimb (G), hindlimb (H), at HH26 in otic vesicle (I), gut (J), forelimb (K), hindlimb (L) at HH28 in retina (M), forelimb (N), and hindlimb (O). o, otic vesicle; b, brain; s, somite; ba, branchial arch; e, eye; np, nasal primordia; h, heart; fl, forelimb; hl, hindlimb; el, eye lens; g, gut; r,retina; asterisk; phalange forming region (PFR).

*Cadherin 11* (*Cdh11*) is also known as osteoblast cadherin. Overall this cadherin shows a very low level ubiquitous expression during early developmental stages and the expression level goes down dramatically during later stages of development. At HH18 it is detected in the brain (b), somites (s) and otic vesicle (o) (Fig. 5A). Expression of *Cdh11*is also detected in the branchial arches (ba), however, major parts of eye (e) including lens and retina, heart (h) and nasal primordia (np) are completely devoid of it (Fig. 5B).Expression is also detected in the limbs (fl, forelimb; hl, hindlimb in Figs. 5C,D). At HH22 the expression spreads to wider domain in the eye (e) with the exception of the lens (el) (Fig. 5E) while expression is detected in the branchial arches (ba) (Fig. 5F) and limbs (fl, forelimb; hl, hindlimb Fig. 5G,H). By HH26, the overall expression level goes down dramatically. However, expression is still detected in otic vesicle (o) and amongst the internal organs, gut (g) is the only structure showing expression (Fig. 5I,J). Limbs still show ubiquitous expression (fl, forelimb; hl, hindlimb in Fig. 5K,L). By HH28 a narrow band of expression is seen in the eye, which marks the retina, (r in Fig. 5M). Expression of *Cdh11* is also detectedin the limbs (Fig. 5N–O). In the hindlimb the expression gets restricted to the phalanx forming region (asterisk in Fig. 5O).

### 3.6 *Cadherin 13*

**Figure 6:**
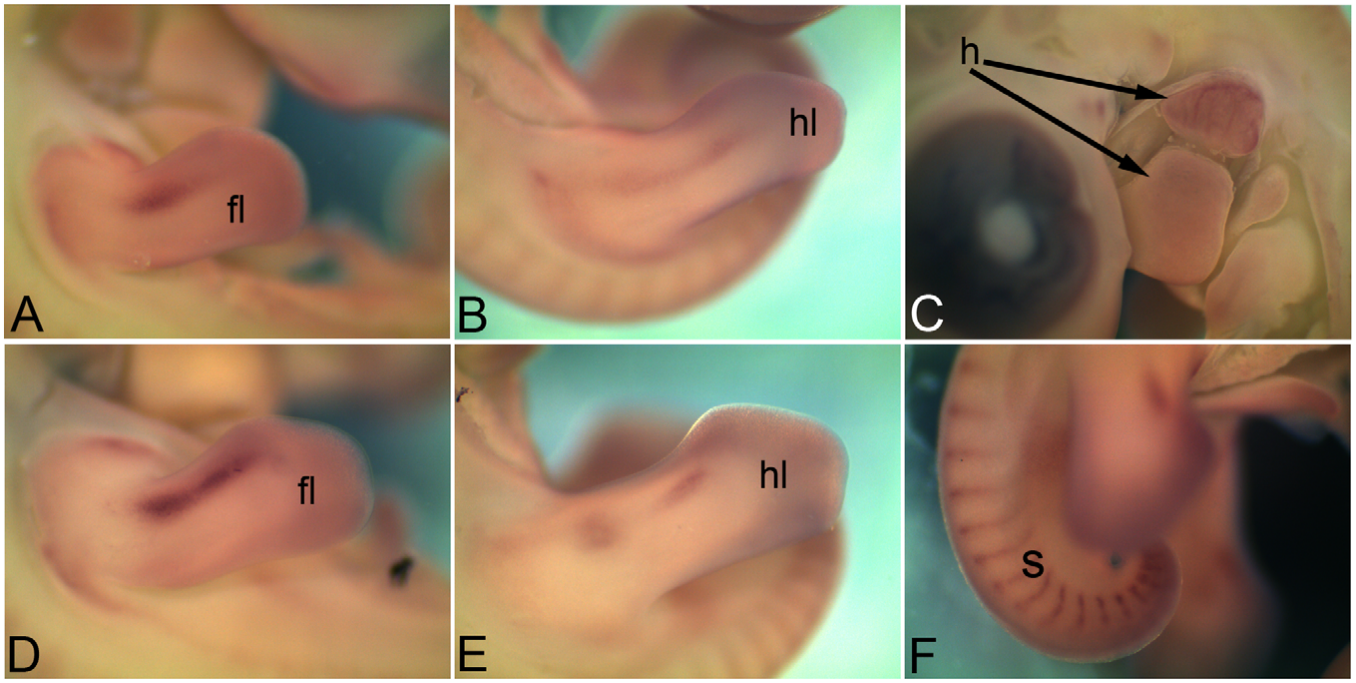
mRNA expression profile of *cadherin 13*. Expression of *Cdh13* at HH26 in forelimb (A), hindlimb (B), heart (C), at HH28 in forelimb (D), hindlimb (E) and caudal somites (F). fl, forelimb; hl, hindlimb; h, heart; s, somite.

*Cadherin 13* (*Cdh13*) is also known as heart-specific cadherin or truncated cadherin. Through whole mount *in situ* hybridization *Cdh13* expression could be detected only at HH26 and HH28. At HH26 the expression domains in the limb (fl, forelimb; hl, hindlimb in Fig. 6A,B) and heart (h in Fig. 6C) were very distinct. At HH28 limb expression domains are still detectable (fl, forelimb; hl, hindlimb in Fig. 6D,E). Further, low level of expression was also detected in somites (s), more in caudal somites, (Fig. 6F).

### 3.7 *Cadherin 23*

**Figure 7:**
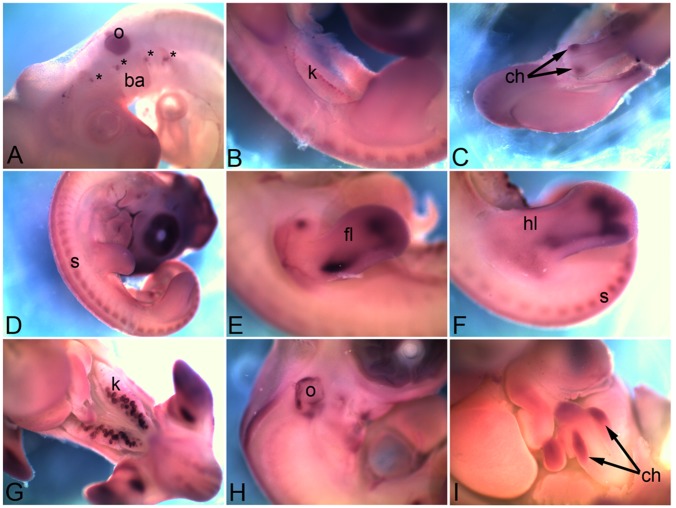
mRNA expression profile of *cadherin 23*. Expression of *Cdh23* at HH18 in otic vesicle and branchial arch (A), at HH22 in kidney (B), caecum (C), somites (D), at HH26 in forelimb (E), hindlimb (F), kidney (G), otic vesicle (H) and caecum (I). o, otic vesicle; ba, branchial arch; k, kidney; ch, caecal horns; s, somite; fl, forelimb; hl, hindlimb; k, kidney; asterisk, base of branchial arch.

*Cadherin 23* (*Cdh23*) is also known as the otocadherin. At HH18 it is expressed in the otic vesicle (s) and in small puncta at the base of branchial arches (asterisk in Fig. 7A). By HH22 it is also expressed in renal glomeruli within the kidney (k) (Fig. 7B), caecal horns in the caecum (ch in Fig. 7C), and somites (s in Fig. 7D). At HH26 it distinctly marks the cartilage domain within limbs (fl, forelimb; hl, hindlimb in Fig. 7E,F). The kidney (k) expression gets distinct within the renal glomeruli (Fig. 7G). The otic vesicle (o in Fig. 7H) expression domain is maintained at HH26. By HH28 expression is lost other than very light expression in caecal horns (ch in Fig. 7I).

### 3.8 *Protocadherin 1*

**Figure 8:**
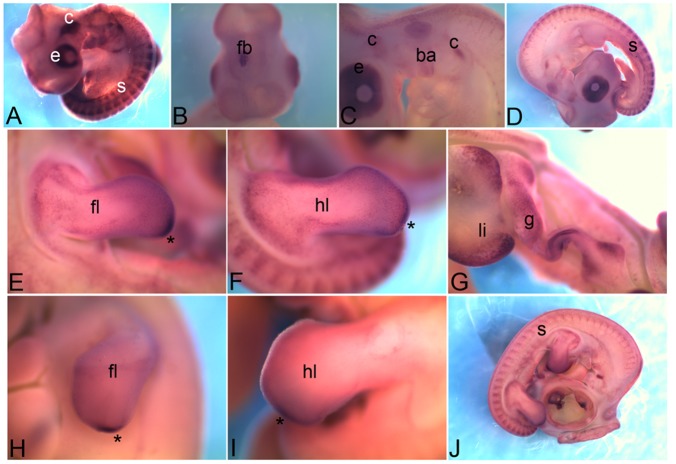
mRNA expression profile of *protocadherin 1*. Expression of *Pcdh1* is detected at HH18 in eye, somite, cranial ganglia (A), forebrain (B), at HH22 in second branchial arch, cranial ganglia (C), somites (D), at HH26 in forelimb (E), hindlimb (F), liver and gut tube (G), at HH28 in forelimb (H), hindlimb (I) and somites (J). e, eye; c, cranial ganglia; s, somite; fb, forebrain; ba, branchial arch; fl, forelimb; hl, hindlimb; li, liver; g, gut; asterisk, posterior-distal domain of expression in limb.

*Protocadherin 1* (*Pcdh1*) expression in general has a mesh like texture similar to *Cdh5*. At HH18 its expression is detected in somites (s), eye (e) and cranial ganglia (c) (Fig. 8A). A unique expression domain within the forebrain (fb) can be seen which might be the cortical hem (Fig. 8B). At HH22 it is expressed in branchial arches (ba) with particularly high level of expression in the second branchial arch, cranial ganglia (c) (Fig. 8C) and somites (s in Fig. 8D). At HH26, expression is also detected in the limbs (fl, forelimb; hl, hindlimb in Fig. 8E,F), liver (li) and gut (g) (Fig. 8G). Within limb there is a small domain at the posterior distal tip which shows an intense expression (asterisk in Fig. 8E,F). At HH28 it is the limb posterior-distal domain of expression that is retained (asterisk in Fig. 8H,I), along with somite (s) while expression in other regions is extinguished (Fig. 8J).

### 3.9 *Protocadherin 8*

**Figure 9:**
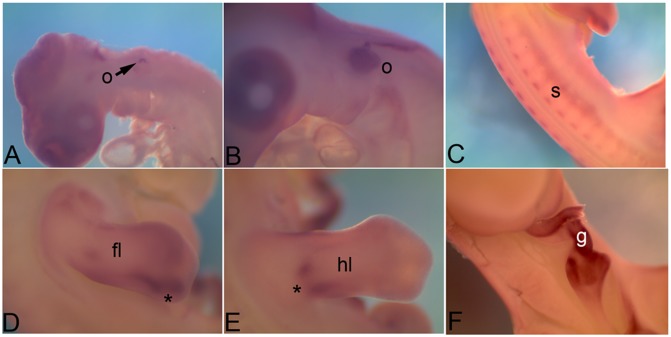
mRNA expression profile of *protocadherin 8*. Expression of *Pcdh8* is detected in otic vesicle at HH18 (A), and HH22 (B), at HH26 in somites (C), in forelimb (D), hindlimb (E) and gut tube (F). o, otic vesicle; s, somite; fl, forelimb; hl, hindlimb; g, gut, asterisk; limb expression domains.

*Protocadherin 8* (*Pcdh8*) is expressed in the otic vesicle (o) at HH18 (Fig. 9A) and HH22 (Fig. 9B). It is expressed in somites (s in Fig. 9C), distinct domains on the posterior end of limbs (asterisk in Figs. 9D,E) and gut (g) at HH26 (Fig. 9F).

### 3.10 *Protocadherin 12 and Protocadherin 18*

**Figure 10:**
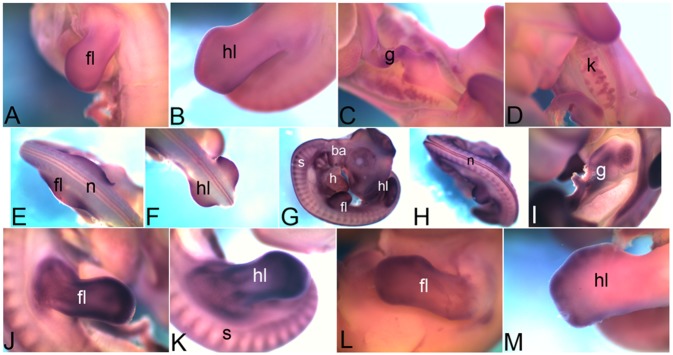
mRNA expression profile of *protocadherin 12* and *protocadherin 18*. Expression of *Pcdh12* is detected at HH26 in forelimb (A), hindlimb (B), gut (C) and kidney (D). Expression of *Pcdh18* at HH18 detected in forelimb and neural tube (E), hindlimb (F), at HH22 in somites, branchial arches, heart, forelimb and hindlimb (G), neural tube (H), at HH26 in gut tube (I), forelimb (J), hindlimb (K), and at HH28 in forelimb (L) and hindlimb (M). fl, forelimb; hl, hindlimb; g, gut; k, kidney; n, neural tube; ba, branchial arch; s, somite; h, heart.

For *protocadherin 12* (*Pcdh12*), expression was detected only at HH26. It was detected to be expressed as a thin band at the periphery of limb buds (fl, forelimb; hl, hindlimb in Figs. 10A,B), the gut (g) and kidney (k) tubules (Figs. 10C,D). *Protocadherin 18* (*Pcdh18*) is expressed at HH18 in neural tube (n) and limb buds (fl, forelimb; hl, hindlimb) (Fig. 10E,F). At HH22 it is detected in somites (s), branchial arches (ba), heart (h), limbs (fl, forelimb; hl, hindlimb) (Fig. 10G) and neural tube (n in Fig. 10H). At HH26 it is detected in gut (g in Fig. 10I) and the limbs (fl, forelimb; hl, hindlimb in Fig. 10J,K). By HH28 all expression domains other than the one in the limb (fl, forelimb; hl, hindlimb in Fig. 10L,M) are lost.

## Discussion

In several developmental contexts cadherins and protocadherins are known to play critical roles. However, our understanding of functional importance of these molecules during embryonic development is far from being comprehensive. Information regarding spatio-temporally restricted domains of expression of a given molecule provides the context in which such studies may be undertaken. The spatio-temporally restricted domains of expression of cadherins and protocadherins uncovered in this study provide such contexts in which the functional roles of these molecules may be investigated.

**Figure 11:**
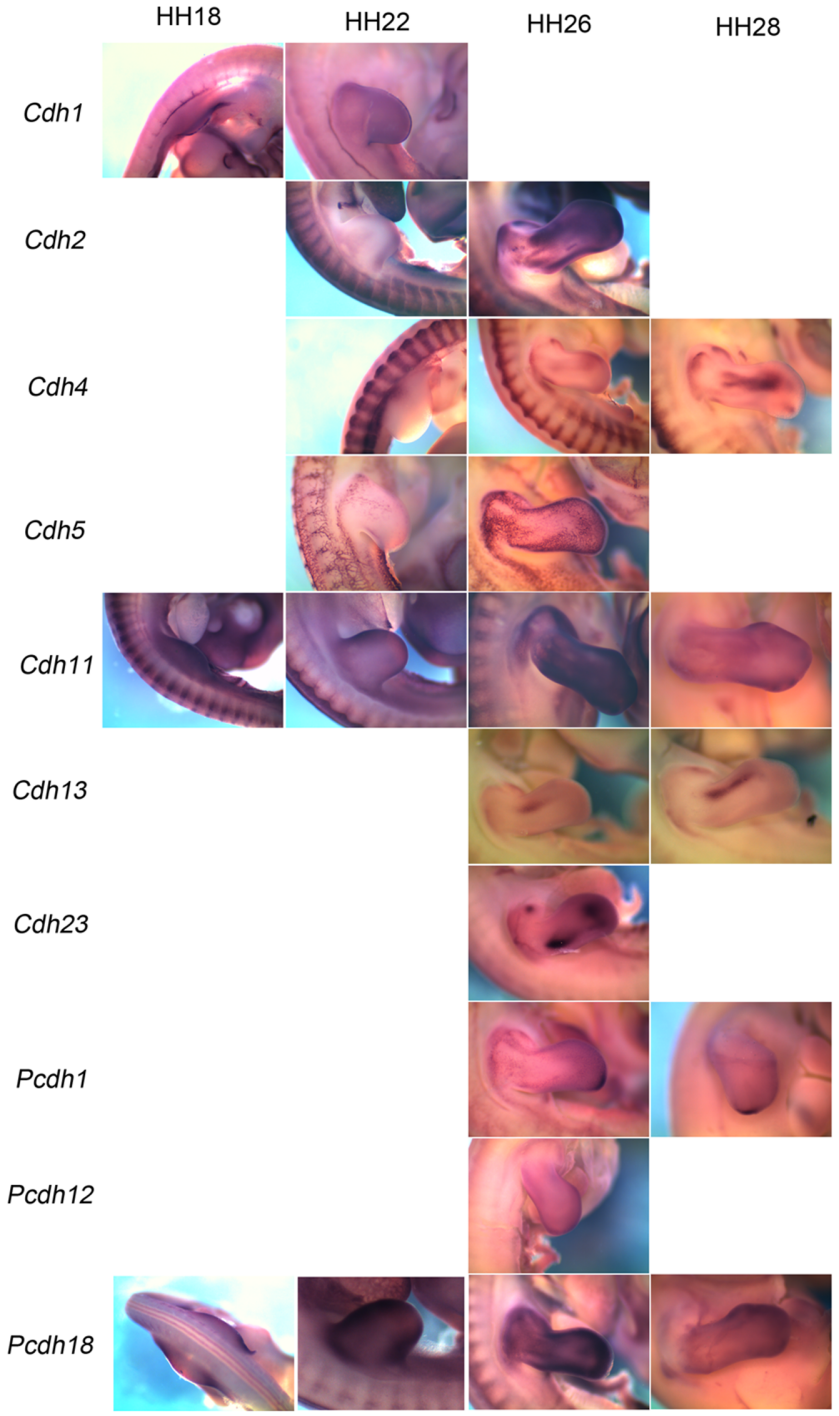
Comparative mRNA expression of different cadherins in the forelimb. Expression of different cadherins detected in forelimb is compared stage-wise. At HH18 *Cdh1*, *Cdh11* and *Pcdh18* are expressed in the forelimb. At HH22 *Cdh1*, *Cdh2*, *Cdh4*, *Cdh5*, *Cdh11* and *Pcdh11* are expressed in the forelimb. At HH26 all cadherins studied in this screen other than *Cdh1* are expressed in the forelimb. At HH28 *Cdh4*, *Cdh11*, *Cdh13*, *Pcdh1* and *Pcdh18* are expressed in the forelimb.

At present most of these molecules are named after the tissue wherein the expression of the gene was first observed. For example, *Cdh1* is also known as the epithelial cadherin, *Cdh2* is also known as the neural cadherin, *Cdh4* as the retinal cadherin etc. However, our expression screen, conducted during the course of embryonic development, reveals that these molecules are in fact expressed in many more and different tissues. For example, *Cdh11* which is known as osteoblast cadherin is also expressed in the brain, somites, branchial arches, otic vesicles, retina etc.. Role of *Cdh11* as tumor suppressor has been established in the context of brain [29] and retina [30]. Thus, osteoblast cadherin might be playing important roles in other tissues where it is shown to be expressed in our study. In the context of limb, the expression is observed as early as HH18, when no osteoblast cells are known to be present. This indicates the possibility that *Cdh11* functions in some non-osteoblast cell types as well during early stages of development. Similarly, comparative expression analysis through this study shows that though all cadherins screened are expressed in the limbs during this developmental time window, each one of them, other than *Cdh11*, is expressed in a different domain within the limb. For easier comprehension we present a collage of panels taken from these figures to demonstrate the varying patterns of expressions of CAMs in forelimb during embryonic development as Fig. 11.

Our expression data when interpreted in the light of existing literature may allow one to form testable hypotheses regarding the function of these genes in different tissue contexts. A good example at hand is *Cdh23*, studied in this screen.

The inner ear of vertebrates and the lateral line in fishes have a specialized sensory tissue, the hair cells. These cells mediate hearing through mechanotransduction i.e., conversion of a mechanical stimulus into electrical signal, which is then transmitted to the central nervous system and processed [31], [32]. On apical surface, hair cells have a unique structure called the hair bundle. Hair bundle is a collection of stereocilia, an actin filled structure surrounded by a membrane, which are heavily cross-linked. The tip of each stereocilium is connected to the lateral membrane of next taller stereocilium through an extracellular filament called tip link [33]. Deflections in hair bundle resulting from mechanical stimulus create tension in a gating spring that opens the mechanoelectrical transduction channels of the hair cells. The resulting depolarization of hair cells results in release of neurotransmitters from the base of hair cells conducting the excitation of hair cells to the CNS.

Waltzer (v) is a genetic locus on mouse chromosome 10 associated with hearing loss. Palma *et al.* identified a new gene mutated in v and named it as otocadherin [34]. *Cdh23* was subsequently shown to be expressed in neurosensory epithelium and essential for hair bundle formation. In mutants, stereocilia organization is disrupted affecting hair-cell differentiation. Recently Siemens *et al* have shown that Cdh23 is one of the components of the adult stereociliary tip links [35]. They propose that in adult hair cells Cdh23 forms the tip links that mediate force transmission to mechanically gated ion channels. Thus they speculate that Cdh23 plays dual role. During development it maintains hair bundles while in the adult hair cells it forms the tip links.

Thus Cdh23 in the context of inner ear is known to play important cilia-related roles be it hair bundle formation or tip links formation. Through these roles it regulates mechanotransduction related activities of hair cells. Through this screen we report that the expression of *Cdh23* is not restricted to inner ear, a component of otic vesicle, but it is also expressed in somites, limbs, kidney, caecum and branchial arches. Though every cell is known to be ciliated, in the context of somite, limb and kidney [36], [37] the function of cilia is better characterized.

Ong *et al* speculated the involvement of flow-sensing cilia, as mediator of “glomerulo-tubular balance” in the context of kidney [38]. Glomerulo-tubular balance refers to the phenomenon which ensures that the rate of fluid re-absorption in the proximal renal tubule is always in proportion to the rate of glomerular filtration [39]. This requires the presence of a mechanotransducer which shall transduce the mechanical stimulus of fluid flow into signals regulating transport across ion channels. Indeed Praetorius and Spring through studies on Madin-Darby canine kidney (MDCK) cells, a canine kidney cell line derived from the collecting duct, reported presence of primary cilia in renal epithelium [40]. Their study showed that this cilia is capable of sensing mechanical forces i.e, it bends when suction is applied with micropipette or if the flow rate of perfusate is increased. It also responds to the mechanical stimulus as indicated by increased calcium uptake. Further this response is not restricted to one cell. It propagates throughout the epithelial tissue by cell-cell interaction mediated by calcium signaling at cellular junctions. This was validated using gap junction permeation inhibitor heptanol which resulted in significant reduction in spread of the response. Thus it is very likely that similar to inner ear [41], renal epithelial cells must have a sensory system capable of sensing the variations in fluid shear stress, amplifying it and transmitting it to the intracellular cytoskeleton and thus mediating the functional regulation of membrane transport proteins. *Cdh23* is a good candidate to be involved in this process.

The limb expression domain of *Cdh23* (Figs. 7E,F) is very similar to the expression of another gene *Papss2* reported in our metabolism related genes (MRG) screen [42] as well as to *Sulf1* as reported by Zhao *et al* [43]. In our MRG screen at HH26 and HH28, *Papss2* is expressed in the condensing mesenchyme [42]. Zhao *et al* through *in situ* hybridization screen in quail embryos showed that at around HH27, *Sulf1* is also expressed in the condensing mesenchyme. In the context of limb skeletal development, mesenchymal cells of the developing limb bud condense and undergo chondrogenesis i.e., differentiation into cartilage fate. This cartilage anlagen serves as the template for bone formation during endochondral ossification. During this process, cartilage cells at the centre of the skeletal element undergo hypertrophic differentiation, followed by matrix secretion and apoptosis. Concomitant to this, vascular, neuronal and osteoblast invasion takes place in this zone followed by ossification which is marked by the formation of bone collar. Hypertrophic differentiation of cartilage is initiated in the middle of the anlagen and spreads outward while cartilage cells near the extremities continue to proliferate and support longitudinal growth of the developing skeletal elements. As the limb skeleton grows, the segmentation of cartilage template takes place. During this process, the incipient joint site loses cartilaginous property and forms a specialized tissue called the interzone. The interzone acts as a signaling centre and instructs the neighbouring cells of the cartilaginous template to achieve distinctive property to become articular cartilage i.e., permanent cartilage. Thus from the condensing mesenchyme of limb bud both bone and articular cartilage are formed. Whitfield referred to the primary cilium of cartilage and bone cells as the mechanosensory toggle switch [44]. In this article Whitfield raises several important questions e.g. given the cilium of cartilage and bone is a mechanosensory device how is it toggled or what is the nature of the signal it sends and how this signaling mode is different from that of other mechanosensing systems. *Cdh23* which our screen reports to be expressed in limb cartilage and from other reports is known to mediate ciliary mechanotransduction might be a good candidate in the above context.

The expression patterns reported herein would thus serve as guidelines to formulate testable hypothesis in different developmental contexts. To elaborate, we would like to use some of the kidney expression patterns elucidated through this study and demonstrate how this expression information can be used in the light of the current understanding of molecular basis of kidney development to develop novel hypotheses.

Kidneys are components of the genito-urinary system. The intermediate mesoderm (IM) generates the entire genito-urinary system including the kidneys. The existing literature suggests that through multiple rounds of sorting of IM cells different structural components of kidney are formed. Cells within these newly formed structures proliferate and differentiate to generate tissues that are capable of supporting the physiological function of kidney [45], [46].

The first morphologically distinct feature arising out of the IM is the nephric duct. As development progresses at the posterior end of the nephric duct a small tissue outgrowth, the ureteric bud, is induced. Tip of the ureteric bud emerging from the nephric duct induces the neighbouring mesenchyme to aggregate around itself and form a distinct structure called the cap mesenchyme. The cap mesenchyme is the source of all epithelial components of the nephron. Cells of the cap mesenchyme aggregate, proliferate, lose their mesenchymal properties and acquire epithelial character through mesenchymal-to-epithelial transition. Changes in cell adhesion properties are known to partly regulate aggregation and epithelialisation of cap mesenchyme cells. At molecular levels *Chd-4* and *Chd-6* expression is upregulated with a concomitant loss of *Cdh-11* expression [47]–[49]. Similarly, Vestweber *et al* have shown that *Cdh-1* is also expressed in the cap mesenchyme, but as development progresses, it’s expression gets restricted to certain segments of renal tubules [50]. The cadherin expression data from this screen are in agreement with the earlier reports describing the expression patterns of *Cdh1*, *Cdh2*, *Cdh4* and *Cdh11* in kidney. However, we are also reporting expression of *Cdh5*, *Cdh23*, *Pcdh1* and *Pcdh12* in developing kidneys.

**Figure 12:**
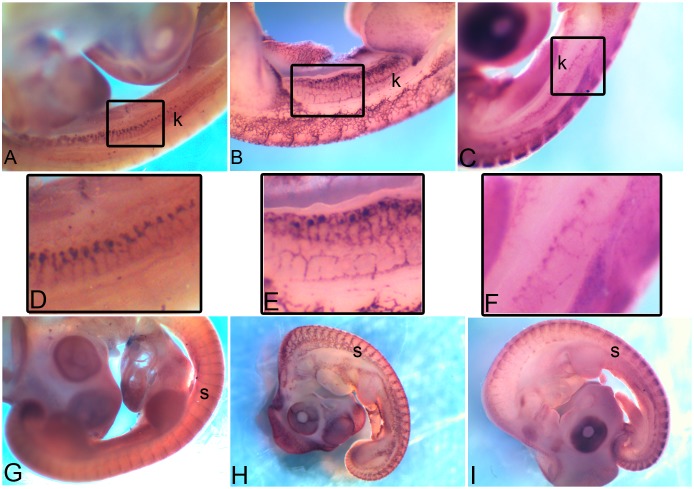
Vascular endothelial-like expression of CAMs. Expression of *Kdr*, *Cdh5* and *Pcdh1* marking kidneys (A, B, C, D, E, F) and somites (G, H, I) respectively at HH22.The rectangular region marked in A, B, C is enlarged in D, E, F respectively. k, kidney; s, somite.

*Cdh5* is the vascular-endothelial cadherin and at HH22 its expression in kidney (k in Fig. 12B) closely resembles the expression of *Kdr* (k in Fig. 12A), a marker of endothelial cells.

The expression patterns of *Pcdh1* (Fig. 12I) is distinct from that of *Cdh5* (Fig. 12H) in other developing structures like the somites (s), however, in the developing kidney *Pcdh1* (k in Fig. 12C, F) is expressed in a pattern similar to *Cdh5* (k in Fig. 12B, E) which in turn is similar to the expression pattern observed for *Kdr* (k in Fig. 12A, D). This raises the possibility that *Cdh5* and *Pcdh1* are working together in the context of renal vasculature morphogenesis. *Pcdh1* (Fig. 12I) is mostly expressed in neuronal structures. thus raising the alternative possibility that *Pcdh1* marks the neuronal cells which are known to closely follow the tracks laid by vascular cells during development. The similar expression of these cadherins in the context of developing kidney thus encourages investigation into the functional roles of these molecules in the context of kidney morphogenesis. Taken together, these expression patterns implicate new candidate cadherin molecules in the context of kidney morphogenesis.
